# Signs of Warning: Do Health Warning Messages on Sweets Affect the Neural Prefrontal Cortex Activity?

**DOI:** 10.3390/nu12123903

**Published:** 2020-12-21

**Authors:** Clara Mehlhose, Antje Risius

**Affiliations:** Marketing of Agricultural and Food Products, Department of Agricultural Economics and Rural Development, University of Göttingen, Platz der Göttinger Sieben 5, 37073 Göttingen, Germany; a.risius@uni-goettingen.de

**Keywords:** fNIRS, graphical health warnings, health warning messages, prefrontal cortex, shocking images, warning label

## Abstract

In the global attempt to combat rising obesity rates, the introduction of health warning messages on food products is discussed as one possible approach. However, the perception of graphical health warning messages in the food context and the possible impact that they may have, in particular at the neuronal level, have hardly been studied. Therefore, the aim of this explorative study was to examine consumers’ reactions (measured as neuronal activity and subjective reporting) of two different types of graphical health warning messages on sweets compared to sweets without warning messages. One type used the red road traffic stop sign as graphical information (“Stop”), while the other one used shocking pictures (“Shock”), an approach similar to the images on cigarette packages. The neural response of 78 participants was examined with the neuroimaging technique functional near-infrared spectroscopy (fNIRS). Different hemodynamic responses in the orbitofrontal cortex (OFC), the frontopolar cortex (FOC), and the dorsolateral prefrontal cortex (dlPFC) were observed, regions which are associated with reward evaluation, social behavior consequences, and self-control. Further, the health warning messages were actively and emotionally remembered by the participants. These findings point to an interesting health information strategy, which should be explored and discussed further.

## 1. Introduction

The number of overweight people worldwide has been rising continuously. In 2016, 39% of adults were considered overweight and 13% even obese [1]. In 2017, 53% of all adults in Germany were overweight, 16% of them obese [2]. This is associated with an increased risk of developing non-communicable diseases such as diabetes, caries, and cardiovascular diseases [3]. Far-reaching negative consequences for society and health systems might follow [4]. In the global attempt to combat overweight, the introduction of health warning messages on food products is currently under discussion [5].

Although the causes of overweight and obesity are complex and multifaceted, there is consensus that a decisive risk factor for overweight is a high energy, low micronutrient diet, which quickly leads to a positive energy imbalance [6]. A high energy density may be caused, for example, by a sugar-rich diet. In Germany, the daily consumption of added sugar is almost twice the quantity recommended by the WHO [7,8]. Exceeding these limits may sometimes happen intentionally, but it is more probable that people are not aware of their exposure to excessive quantities of sugar and the potential resulting health consequences [9].

The behavioral regulation of eating-related processes is a constant consideration between rewarding food stimuli around us, on the one hand, and self-regulating control processes, on the other hand [10]. Through the omnipresence of tasty and often high-caloric food cues in our society, our desire to eat gets constantly and unconsciously stimulated, which affects our actual behavior more than we think [11]. Food cues trigger areas in the brain that are responsible for reward and taste [12], inducing physiological reactions such as salivation [13], and easily lead to food-cravings and excessive consumption, even if there is no metabolic demand at that moment [14,15]. The rewarding effects of food cues can be suppressed by the self-regulating control system, which makes it possible to resist appetizing food cues and thus to maintain personal long-term goals such as healthy eating or the avoidance of weight gain [10]. However, it seems that food cues have different effects on overweight people than on people with normal weight, in the sense that obese people show increased reward activation [16,17] and decreased self-regulation activation in reaction to different food cues [18,19]. Obesity is also associated with automatic and stronger approach tendencies toward unhealthy food stimuli compared to those of normal-weight people [20,21]. It seems that a poor ability to self-regulate eating behavior and habits and the automatic processes guiding unhealthy eating behavior may prevent people from resisting to rewarding food stimuli [22,23]. As such, food cues and the conditioned reactions of the body to these cues significantly contribute to eating behavior and weight gain [24,25]. Creating cues that help avoid undesired but automatic food-related behavior, such as health warning messages, might trigger a healthy behavior and strengthen self-control [22].

Health warning messages or warning labels are expected to work in two ways: On the one hand, they address the cognitive system in providing information about the health consequences associated with consumption, which can intensify the intention to change this behavior and reduce purchase intentions for unhealthy food products [26,27,28,29]. On the other hand, health warnings are expected to disrupt unconscious influences on food decisions, e.g., food cues and other external triggers, in priming a healthier food decision-making behavior [30,31].

The perception of food warning messages has not yet been sufficiently investigated, even though food warning messages already exist in some countries. The currently used ones, e.g., in Chile, are text-based only [32], although studies from different contexts (e.g., snacks in general, tobacco, alcohol, or sugar-sweetened beverages) all came to the conclusion that graphical health warning messages seem to be more effective than text-based ones [31,33,34,35,36,37,38,39,40,41]. Therefore, we examined graphical health warning messages in this study.

In order to get a better understanding of the possible effects health warning messages might have, it is of great interest to gain more insight into individual perceptions. Basically, one can distinguish two approaches: *Direct approaches* ask people directly about their perception and opinion concerning health warning messages. This helps to understand what people consciously think about it. However, direct questioning is insufficient when it comes to investigating the unconscious reactions and possible indirect effects of warnings as well, because a questionnaire often raises the problem of socially desirable response behavior and the fact that people are not aware of all their emotions, feelings, and so on. To this end, neuroimaging measurements can be a valuable tool, in the branch of *indirect assessments*. With their help, one can observe in which areas of the brain an increased neural activity can be measured in response to various stimuli. How food warning messages are perceived and the neural effects they have on consumers are not yet understood. So far, only one study examined the neural effects of warning messages in the context of food products: Rosenblatt et al. (2018) found out, with the use of electroencephalography (EEG), that health warning messages enhanced dietary self-control through a reduction of the food cue reactivity. The EEG technique used in the above-mentioned study allows for a good temporal resolution of the brain activity data but not for a good spatial resolution. Other common neuroimaging studies use functional magnetic resonance imaging (fMRI), which is often limited to the study of a larger number of subjects because of its cost intensity and regarding the selection of the participants [42]. To tackle obesity and overweight research that involves large parts of society, it is crucial to examine more participants than the typically investigated 25–30 subjects in these studies. To overcome both aspects, we used a novel imaging process technology called functional near-infrared spectroscopy (fNIRS) in this study [43]. It is more cost-effective than fMRI, and there are no restrictions regarding the selection of the participants. It therefore allows for an examination of the involved neural activity through hemodynamic responses in a large number of subjects [44]. This may be helpful in understanding the neural effects of food-related health warning messages in a quantitative manner. However, there are also limitations of fNIRS: There is poorer spatial resolution compared to fMRI due to the fact that fNIRS measurement is based on hemoglobin concentration changes measured through near-infrared light, whereas fMRI is measured through the changes in magnetic fields. In fNIRS, this leads to a low light penetration depth, which means that only the superficial areas of the brain can be measured, such as the prefrontal cortex. This also makes it more challenging to precisely localize the activated areas, as they are difficult to distinguish from one another. This might lead to results that are not as concrete or clearly interpretable to explain the underlying human behavior, which is why we combined the approach with a questionnaire about participants’ health behavior, including a subjective reporting about the perception of the products with the health warning messages. Understanding the perception of health warning messages seems critical when there is a worldwide discussion about implementing such labels to pave the way for health strategies against non-communicable food-related diseases such as obesity and diabetes.

The purpose of this study was to examine consumers’ perception of two different types of graphical health warning messages on sweets. One type used the red road traffic stop sign as graphical information, as it is a sign used in traffic known worldwide. Further, there already exist food warnings that have the form of stop signs, e.g., in Chile. The other one used shocking pictures (alternatingly one of a denture affected by caries and one of a foot with diabetic foot syndrome), an approach similar to that used in shocking images on cigarette packages. Both types of graphical messages were supplemented by textual information about caries or the diabetic foot syndrome. The aim of this paper was twofold: On the one hand, we wanted to investigate the neural responses of participants with fNIRS as measured by hemodynamic activities in prefrontal brain regions during the perception of these health warning messages, in order to explore which brain areas and related psychological processes might be important to enhance the understanding and effectiveness of graphical warning signs. On the other hand, we aimed to compare how the two types of warning messages were perceived by the participants, which we did by analyzing their subjective reporting in a direct assessment and by contrasting them with the fNIRS measurements. Due to the novelty of fNIRS as an imaging technology for examining consumer behavior, this study is of an explorative character, further aiming to validate this methodology against other elements of socio-empirical research.

## 2. Materials and Methods

### 2.1. Participants

A hundred and three German students were randomly recruited to take part in the study. All of them gave their written consent to participate in this study and received monetary compensation at the end of the study (5€). They were all informed about the test procedure, the fNIRS methodology, and that they could withdraw from the study at any time without consequences. The study was conducted in accordance with the Declaration of Helsinki and with the principles of the ICC/ESOMAR Code. The procedure and the use of fNIRS were approved by the Ethical Committee of the University. Twenty-five participants had to be excluded due to poor data quality (22 participants) or errors during data recording (three participants). Data analysis was hence continued with 78 participants. Of these, 44 were female and 34 of them male. The mean age was 25.4 years, with ages ranging from 18 to 39. The average body mass index (BMI) was 23.4 and ranged from a minimum of 15.8 to a maximum of 37.5. For further analysis, we divided the participants into two groups according to their BMI. The High_BMI group consisted of 22 persons, 10 men and 12 women, who had a BMI classified as overweight or obese (cut-off value men: 25, cut-off value women: 24). The upper and lower limits of the BMI value classes are slightly higher for men than for women (the WHO recommends 25 for men and women) because men generally have a higher proportion of muscle mass in total body mass than women. Their mean age was 26 years. We checked for significant differences of age between the two groups but found no differences. The Normal_BMI group consisted of 56 participants, of which 24 were men and 32 were women, and the mean age of this group was 25.1 years. The High_BMI group was considerably smaller, since we could not explicitly recruit the subjects based on their body weight/BMI due to ethical reasons.

### 2.2. Stimuli

To create the health warning messages, we used two different types of graphical symbols: First, we used the red traffic road stop sign as a graphical warning symbol (“Stop”). Then, we used shocking images as graphical warning symbols, similar to those on tobacco packages (“Shock”). For this, we used a picture of a denture affected by caries and a second picture with a foot affected by diabetic foot syndrome. We selected these diseases to illustrate the effects of caries and diabetes, because they are well-known secondary diseases of excessive sugar consumption [45].

Further, we used two different types of textual information to combine them with the graphical symbols: one provided information about caries and the other one about the diabetic foot syndrome. The information about caries read (translated from the German original): *“Caries (lat. rottenness, morass) is a multifactorial dental disease that is promoted by sugar”*. Similarly, the information about the diabetic foot syndrome read: *“Excessive sugar consumption leads to tooth decay, obesity, and diabetes. The diabetic foot is a common secondary disease of diabetes mellitus”.*

**Figure 1:**
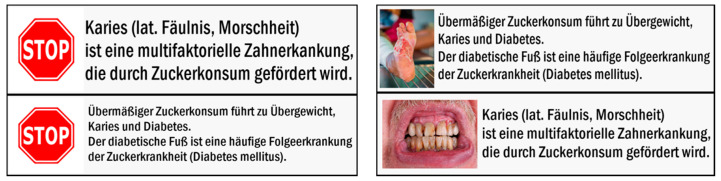
Four different health warning messages were created. For the “Stop” examination condition, a stop sign was used (two pictures on the left). For the “Shock” examination condition, two shocking pictures (one of a denture affected by caries and one of a foot with diabetic foot syndrome) were used. The textual information informed about caries and the diabetic foot syndrome and was identical for both types of warning messages.

So, in total, four different health warning messages were created, each consisting of a graphical symbol and the appropriate textual information next to it. This resulted in a 2 (“Stop”/”Shock”) × 2 (caries/diabetic foot syndrome) factorial design. The graphical and textual warnings are shown in Figure 1.

The warning messages were printed on small stickers (21 × 76 mm) and then attached onto real sweets products. We used eight different sweets products of the type gummy bears, chocolate bars, and cookies. Each product was labeled once with each warning message. In total, this resulted in 32 differently labeled sweets (four different graphical warnings each on eight different products). All products were bought in a typical German grocery store, and all items were branded products to ensure that they were familiar to the participants. Our aim was to present the products as realistically as possible. Therefore, pictures of all products were then taken to present them as visual stimuli to the participants. The images were edited with the help of the image-editing program Paint 3D but only to remove shadows or reflections.

To conserve statistical power, the examination conditions of the experimental design differed only between the two different graphical types of health warning messages. This means that all textual health warning variants (caries/diabetic foot syndrome) were grouped together, resulting in a “Stop” condition and a “Shock” condition. This resulted in 16 different pictures for each of the two experimental conditions. Additionally, we used seven other unlabeled sweet products to have a wider range of products for a neutral control condition, resulting in 15 pictures for the control condition (the same eight different products from the examination condition unlabeled, plus seven additional products, also unlabeled).

### 2.3. Experimental Design

**Figure 2:**
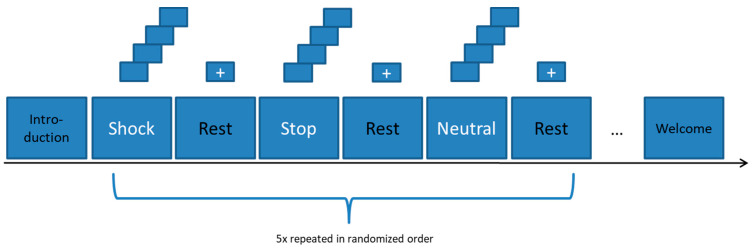
Experimental Design of the fNIRS experiment. A block design with three experimental conditions (“Shock”, “Stop”, and “Neutral”) was used. The order of the trials was randomized.

The fNIRS experiment consisted of a block design with two experimental conditions (“Stop” condition and “Shock” condition) and one control condition (“Neutral” condition). In total, there were three different blocks with five runs per block. The order of the blocks was randomized. One block consisted of four randomly chosen pictures from the respective condition. Each picture was shown for 5 s, resulting in a duration of 20 s per block, followed by a pause of 25 s, which was represented by a small cross on the screen. The presentation started and ended with instructions, which were shown for 9 s each. This lasted for a total experimental duration of 11.5 min. The precise instructions to the participants were as follows: “Please look at the products carefully and pay attention to noticeable differences.” The experimental design can be seen in Figure 2.

### 2.4. Study Procedure

Each participant completed a single experimental fNIRS session, followed by a questionnaire. The study took place in a room specifically set up for the experiment, which contained no furniture or wall decoration other than a table and chair for the subject and the experiment leader. It was also ensured that no direct light fell on the fNIRS setup. The fNIRS experiment and the questionnaire were computer-based and were presented to the subjects on a 19.4-inch computer screen. First, the participants were informed about the basic procedure of the study and had the opportunity to ask questions before agreeing to participate. They were then fitted with the fNIRS headband and asked to sit as still as possible and to avoid strong head movements for the duration of the experiment. We then started the presentation of the stimuli. After completing the fNIRS part of the experiment, the participants’ headband was removed again, and they were asked to respond to the questionnaire. The stimulus presentation and the experimental control were carried out with the help of the program Presentation (version 20.3.), by the company NeuroBehavioralSystems (NBS). The survey data was collected through the online survey software program Unipark, from Questback.

### 2.5. fNIRS Measurement and Data Analysis

**Figure 3:**
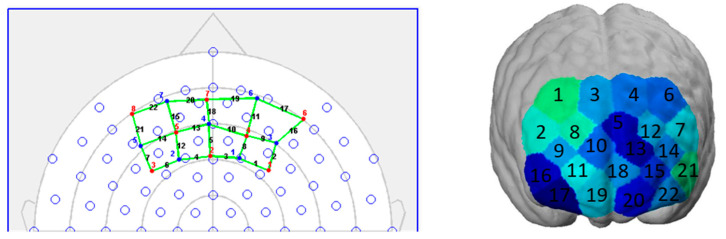
(**Left**) Topographical layout of the NIRx headband. The red numbers (1–8) point out the placement of the light sources, and the blue numbers (1–7) the placement of the light detectors. One source and one detector result in one measurement channel, visualized through the black channel numbers (1–22). The reference points refer to the EEG 10–20 system. (**Right**) The coverage of the headband is shown on the ICBM 152 head model. The color shadings do not have any meaning here.

fNIRS visualizes brain activity through the ability of hemoglobin to absorb light. Sources of near-infrared light penetrating the human tissue are used to measure the differences of cerebral oxygenated hemoglobin (oxy-Hb) and deoxygenated hemoglobin (deoxy-Hb) at certain times [46]. We can therefore draw conclusions about the neural activity beneath the surface of the brain: An increase in metabolic demand for oxygen during a mental task leads to a stronger cerebral blood flow in this area, to fulfill the demand. This so-called “neurovascular coupling” principle is quantified in fNIRS through the measurement of the absorption rate of the different colors of blood (brighter red = oxygenated blood, darker red = deoxygenated blood) [44]. As such, the mechanism is similar to the principle used in fMRI measurements (BOLD signal), which allows the fNIRS signal to be compared to the fMRI signal [47]. The advantage of fNIRS, however, is that due to its simple setup, it is much more cost-effective and mobile than fMRI [48]. As the fNIRS setup is non-invasive and only works with near-infrared light, there are no safety concerns, which makes fNIRS suitable for all types of participants and permits an analysis of larger sample sizes [44]. We used a mobile fNIRS device (NIRSport, NIRx Medical Technologies, Berlin, Germany) for the measurements in this study. This is a two-wavelength (760 and 850 nm) continuous wave system that collects data in parallel at a sampling rate of 7.81 Hz. On a neoprene headband, eight light sources and seven light detectors are placed at a distance of three centimeters of each other. This results in 22 measurement channels covering parts of the prefrontal cortex. Specifically, it covers parts of Brodmann Area 9, 10, 11, and 46, and thus parts of the orbitofrontal (OFC), the dorsolateral (dlPFC), and the frontopolar prefrontal cortex (FPC). A schematic representation of the topographical layout (left) and the coverage on a brain map (right) are shown in Figure 3.

The headband was placed on the forehead of each participant according to the craniometric points of the head. We used the nasion of each participant as a measurement point to place the source number 7 in the same place for each participant, and thus created comparability between the participants. NIRStar software package (version 15.0) was used to check for signal quality and data collection.

Before analyzing the data, we performed several preprocessing steps of the raw optical data: First, we verified the data quality of every individual dataset. We compared the signal-to-noise ratio of each channel with the coefficient of variation (CV). Channels with a value higher than the threshold of 7.5 (default value) were marked as bad and excluded from the subsequent analysis. Participants with half of all or more bad channels were excluded from further analysis. Twenty-two participants were completely removed for this reason. We then band-pass filtered the data (of 0.01–0.2 Hz) to smooth out both very rapid and very slow fluctuations. Strong motion artifacts and discontinuities were removed manually as well. The raw optical density signals were then modified into hemoglobin concentration changes through a modified Beer–Lambert law. In terms of parameters, the wavelength was specified to values of 760 and 850 nanometers, and the pathlength factors were set to 7.25 and 6.38. Data analysis was continued only with oxy-Hb, as this has been shown to correlate more strongly with the cerebral blood flow [49]. The preprocessing of the raw optical data as well as the analysis of the neural data were performed with the NIRx Software package nirsLAB version 2019.4.

We analyzed the hemodynamic-state time series of the different channels during the experimental conditions with the help of a General Linear Model (GLM) for every participant and each block. We modeled the parameters of the single experimental trials grouped together, resulting in three event-related regressors and the constant error term (Y_j_ = x_j1_β_1_ + x_j2_β_2_ + x_j3_β_3_ + ε_j_). To model the onsets of neural activity, we used the hemodynamic response function. T-contrasts were calculated for the main effects of the single conditions and to compare the experimental conditions with the control condition (Stop vs. Neutral, Shock vs. Neutral). These contrasts were used to generate statistical parametric maps (SPM) for the whole group of participants (between-subject level). Further, we contrasted two subgroups of the sample based on their physical constitution (measured with BMI) in relation to the experimental conditions (HighBMI_Shock vs. NormalBMI_Shock, HighBMI_Stop vs. NormalBMI_Stop, NormalBMI_Shock vs. HighBMI_Shock, NormalBMI_Stop vs. HighBMI_Shock). The activation threshold was set at least to *p* < 0.1, and the thresholded brain activation maps were then plotted on the standardized head model (ICBM-152), which is included in the nirsLAB software. We matched the fNIRS channels with the 10–20 system EEG reference points and mapped them according to [50] and with the help of the fOLD toolbox (v2.2) for information about the activated anatomical areas of the brain. These refer to Brodmann Areas and the brain regions associated with them. Multiple comparison corrections were performed using the false discovery rate (FDR) method.

### 2.6. Direct Assessment (Questionnaire)

Immediately after the end of the fNIRS experiment, the participants were asked to fill in a questionnaire. The first two questions were open questions, where we aimed to determine whether the participants recognized the health warning messages on the products, and we asked if they believed that the shown products might influence their purchase behavior and why. With the first question, we aimed to find out whether the participants recognized the health warning messages at all and whether they paid attention to them. The second question aimed to identify their beliefs and intentions concerning the possible effectiveness of the health warning messages, i.e., the influence on their purchase behavior. Furthermore, the questionnaire contained general questions about the food, sleep, and movement behavior of the participants. The evaluation of the open questions was based on [51]. We evaluated the answers of all subjects (excluding one who did not answer this question) who participated in the fNIRS experiment (resulting in n = 102) and not only those who had valid data for the fNIRS analysis. This allowed us to compare the impressions about the general perception of health warning messages.

## 3. Results

We observed different hemodynamic responses regarding the main effects of the experimental conditions, the two different graphical health warning messages compared to the control condition, and the subgroups of the sample based on their physical constitution (BMI). The results of the main effects are reported with a significance level of *p ≤* 0.05, and those of the experimental conditions compared to the control conditions with *p ≤* 0.1. This was done because our conditions did not differ significantly, since we used for both experimental conditions the same product pictures, and only the health warning messages differed, not leading to very strong reactions. The results indicate increased neural activity in brain regions of the prefrontal cortex—among others, of the orbitofrontal cortex (OFC), the frontopolar cortex (FOC), and the dorsolateral prefrontal cortex (dlPFC).

### 3.1. Main Effects of the Experimental Conditions

**Figure 4:**
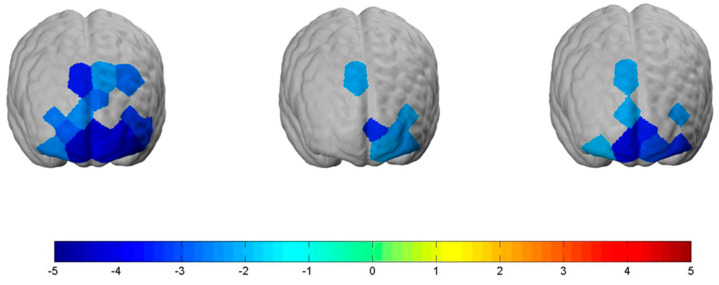
Significantly decreased neural prefrontal cortex activation for the main effects of the “Neutral” (**left** brain), “Stop” (**middle** brain), and “Shock” condition (**right** brain). Activation threshold set to *p* < 0.05.

The main effects of the experimental conditions can be associated with the following results, which are significant on a level of *p ≤* 0.05:

The “Neutral” condition significantly decreased neural activity in parts of the *dlPFC* (*channel 3*: t(78) = −3.56, *p ≤* 0.001, d = 0.8; *channel 4*: t(78) = −2.23, *p ≤* 0.05, d = 0.5; *channel 5*: t(78) = −2.74, *p ≤* 0.01, d = 0.62; *channel 6*: t(78) = −2.79, *p ≤* 0.01, d = 0.63; *channel 9*: t(78) = −2.63, *p ≤* 0.05, d = 0.59; *channel 14*: t(78) = −3.22, *p ≤* 0.01, d = 0.72; *channel 21*: t(78) = −3.23, *p ≤* 0.01, d = 0.73), the *OFC* (*channel 17*: t(78) = −2.45, *p ≤* 0.05, d = 0.55; *channel 19*: t(78) = −4.14, *p ≤* 0.001, d = 0.90; *channel 20*: t(78) = −3.78, *p ≤* 0.001, d = 0.85; *channel 22*: t(78) = −4.51, *p ≤* 0.001, d = 1.0), and the *FPC* (*channel 10*: t(78) = −2.43, *p ≤* 0.05, d = 0.55; *channel 11*: t(78) = −2.96, *p ≤* 0.01, d = 0.67; *channel 15*: t(78) = −3.87, *p ≤* 0.001, d = 0.87; *channel 18*: t(78) = −4.22, *p ≤* 0.001, d = 0.95) (see Figure 4, left brain).

The “Stop” condition significantly decreased neural activity in parts of the *dlPFC* (*channel 3*: t(78) = −2.06, *p ≤* 0.05, d = 0.46; *channel 14*: t(78) = −2.12, *p ≤* 0.05, d = 0.48), the *OFC* (*channel 20*: t(78) = −2.08, *p ≤* 0.05, d = 0.47; *channel 22*: t(78) = −2.2, *p ≤* 0.05, d = 0.5), and the *FPC* (*channel* 15: t(78) = −2.22, *p ≤* 0.05, d = 0.5; *channel 18*: t(78) = −3.4, *p ≤* 0.01, d = 0.7) (see Figure 4, brain in the middle).

The “Shock” condition significantly also decreased neural activity in parts of the *dlPFC* (*channel 3*: t(78) = −2.15, *p ≤* 0.05, d = 0.48; *channel 14:* t(78) = −2.28, *p ≤* 0.05, d = 0.51), the *FPC* (*channel 10*: t(78) = −2.06, *p ≤* 0.05, d = 0.46, *channel 18*: t(78) = −3.53, *p ≤* 0.05, d = 0.79), and the *OFC* (*channel 17*: t(78) = −2.0 *p ≤* 0.05, d = 0.45; *channel 19*: t(78) = −3.71, *p ≤* 0.001, d = 0.84; *channel 20*: t(78) = −3.05, *p ≤* 0.01, d = 0.69; *channel 22*: t(78) = −3.38, *p ≤* 0.01, d = 0.76) (see Figure 4, right brain).

### 3.2. Experimental Conditions Compared to Control Condition

Related to all participants, the contrasts of the experimental conditions (“Stop” and “Shock”) compared to the control condition (“Neutral”) can be associated with the following results, which are all significant on a liberal significance level of at least *p ≤* 0.1.

**Figure 5:**
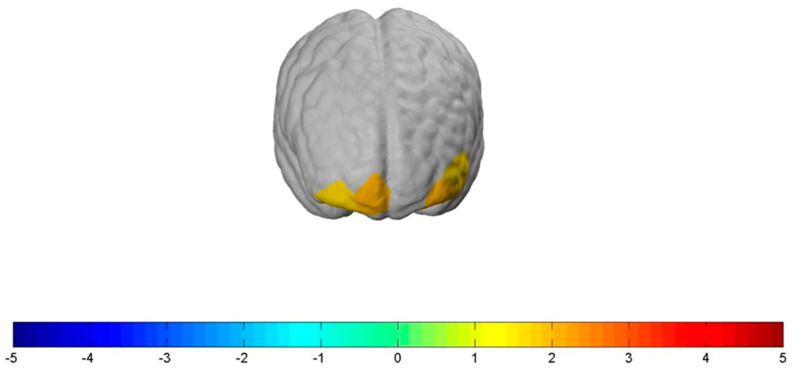
Significantly increased neural prefrontal cortex activation for the contrast between the “Stop” condition versus the “Neutral” condition: channel 22: t(78) = 1.93, *p ≤* 0.1, d = 0.437; channel 21: t(78) = 1.69, *p ≤* 0.1, d = 0.382; channel 19: t(78) = 1.97, *p ≤* 0.1, d = 0.315, and channel 17: t(78) = 1.71, *p ≤* 0.1, d = 0.446. The activation threshold was set to *p* < 0.1.

**Figure 6:**
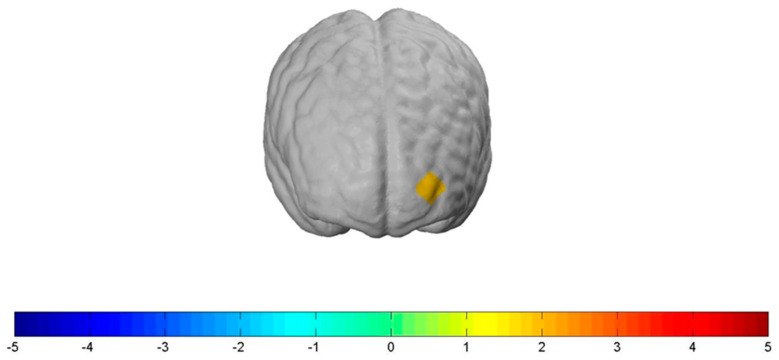
Significantly increased neural prefrontal cortex activation for the contrast between the “Shock” condition versus the “Neutral” condition: channel 15: t (78) = 1.757, *p ≤* 0.1, d = 0.398. The activation threshold was set to *p* < 0.1.

The “Stop” condition significantly increased bilateral neural activity in the OFC and the FPC. More specifically, the “Stop” condition led to a significantly increased bilateral neural activation in *channel 22*: t(78) = 1.93, *p ≤* 0.1, d = 0.437; *channel 21*: t(78) = 1.69, *p ≤* 0.1, d = 0.382; *channel 19*: t(78) = 1.97, *p ≤* 0.1, d = 0.315, and *channel 17*: t(78) = 1.71, *p ≤* 0.1, d = 0.446, when compared to the “Neutral” condition (see Figure 5). However, the “Shock” condition, when compared to the “Neutral” condition, did not show increased neural activity in the OFC but led to a significantly increased lateral neural activation in the FPC, more precisely in *channel 15*: t(78) = 1.757, *p ≤* 0.1, d = 0.398 (see Figure 6). In order to talk about differences between the two types of health warnings, we also contrasted them directly. However, we did not find significant results for the interaction of the “Stop” with the “Shock” condition compared to the “Neutral” one.

### 3.3. Group Comparison of High vs. Normal BMI

**Figure 7:**
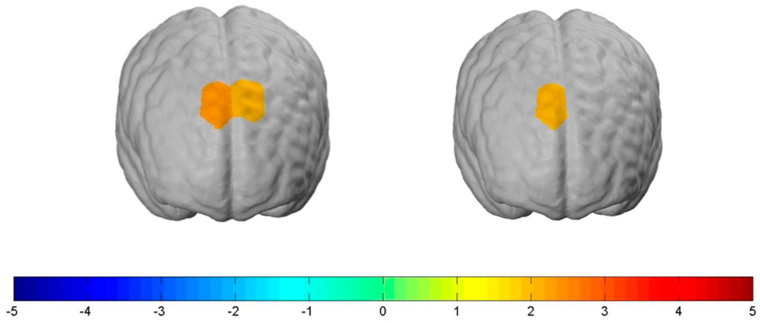
Contrast of the NormalBMI group (n = 56) with the HighBMI group (n = 22) for the two experimental conditions. The participants with a normal BMI showed significantly increased prefrontal cortex activation compared to the ones with a high BMI in both experimental conditions. **Left** brain: For the NormalBMI group, neural activity in the dlPFC increased when exposed to the “Stop” condition. **Right** brain: For the NormalBMI group, neural activity in the dlPFC increased when exposed to the “Shock” condition.

According to the physical constitution of the participants, we contrasted one group with a normal BMI (n = 56) (NormalBMI) and the corresponding hemodynamic response to the experimental conditions with another group with a high BMI (n = 22) (HighBMI) and their neural activity in relation to the experimental conditions. This achieved the following results, which are all significant on a liberal significance level of at least *p ≤* 0.1:

For the NormalBMI group, increased bilateral neural activity in parts of the dlPFC compared to the HighBMI group was measured when exposed to the “Stop” condition. More specifically, this held true for *channel 3*: t(78) = 2.19, *p ≤* 0.05, d = 0.53 and *channel 4*: t(78) = 1.90, *p ≤* 0.1, d = 0.26. Further, we also measured increased lateral neural activity in the dlPFC when exposed to the “Shock” condition. This held true for *channel 3*: t(78) = 1.90, *p ≤* 0.1, d = 0.43 (see Figure 7). We found no significant differences between the two groups when being exposed to the control condition.

### 3.4. Direct Assessment (Questionnaire)

We directly asked participants what they focused their attention on during the experiment. The exact question was: “What did you look for?” Altogether, we classified 253 statements and assigned them to four different categories: statements related to the warning messages (100 counts/39.53%), to the products or individual product features (78 counts/30.83%), to the packaging of the products (57 counts/22.53%), and to the change of the focus (18 counts/7.11%). Interestingly, the majority of the participants actively perceived and remembered the health warnings. 75.7% of the participants mentioned that, amongst other categories, they actively paid attention to the health warnings. The other 24.3% provided statements of the other categories only.

Further, we asked whether the participants thought that the products they saw would influence their purchase behavior and why. The exact question was: “What do you think: would the shown products have an effect on your buying behavior? Why?” For this question, we classified 234 statements and assigned them to four different categories: statements related to the effectiveness of the health warnings in general (108 counts/46.15%), statements that mentioned “No influence on my behavior, because…” (66 counts/28.21%), statements that mentioned “Yes, it would influence my behavior, because…” (44 counts/18.8%), and statements related to the influence of the products itself (12 counts/5.13%).

## 4. Discussion

The main focus of this study was on neural prefrontal cortex activation caused by the perception of two different types of health warning messages on sweet snack products.

Our results show that both for the “Stop” condition and the “Shock” condition, participants’ neuronal activity increased significantly compared to the “Neutral” control condition. This is in line with a number of other neuroimaging studies that also found an increase in neuronal activity in relation to graphical and text-based warnings, mostly in the context of smoking behavior [5,52,53,54,55]. To the best of our knowledge, in the context of food products, only one study has examined the neural signals of health warning messages thus far. It found a reduced neural reaction to these messages, which was explained by an automatic appetite-control-regulation of the participants [30]. However, a comparison with our results is only possible to a limited extent, since EEG was used in the other study, an imaging procedure based on the electrical activity of the brain and not based on the metabolic activity of the brain, as is the case with fNIRS or fMRI measurements.

Our results revealed, furthermore, that the “Stop” condition significantly increased activation in parts of the OFC. The OFC plays a crucial role in representing and evaluating positive and negative reinforcers and is considered part of the reward region of the brain [56,57]. As such, it is associated with many cognitive processes in the context of integrating sensory information to subsequent behavior [56,57,58,59,60,61,62,63,64]. Our results are in line with another study, which found increased activation in the OFC for aversive smoking images versus control images [53]. The authors explained their results based on the fact that smoker experienced an arousing and unpleasant emotional response when attending to the negative value of their consumption and therefore try to modulate or suppress these feelings. Hollmann et al. (2012) [65] asked participants to regulate their desire for food and thereby evaluated the long-term consequences of eating the food for society and health. They found an increased hemodynamic response in this area as well and interpreted it as an evaluation of the negative consequences following of the absence of a reward. It is therefore conceivable that these processes are also part of the processing of health warning messages. Our results revealed, further, a significant increase in activation in one channel of the frontopolar cortex (BA 10) for the “Shock” as well as for the “Stop” condition when compared to the neutral condition. This is the only significant neural activation which we found for the “Shock” condition. Basically, this is in line with the results of, e.g., Chua et al. (2009) [66], who found activation in the rostral medial prefrontal cortex (BA 10) for text-based health warning messages. The FPC plays a key role in future thinking and planning and has been associated with emotional, cognitive, and social processes, and especially with the long-term consequences of social behavior as well as feelings of guilt. We can only speculate at this point, but as almost half of all answers of the direct approach were statements related to the effectiveness of the health warnings in general, it is conceivable that the shock warnings in particular are impressive reminders of the consequences of unhealthy behavior or might even cause feelings of guilt.

Further, our results showed that participants with a normal BMI had significantly increased neural activity in the dlPFC in both experimental conditions compared to participants with a higher BMI, who had a significant decrease in neural activity. The dlPFC is part of the prefrontal control region and is, in this context, associated with self-regulation and self-control [67,68,69,70], weighting the immediate rewarding values, e.g., of food cues, against the expected personal long-term consequences (positive or negative) resulting from them [71]. This shows an increased hemodynamic response when regulating processes to suppress food or cigarette cravings [65,69,72]. Our results are therefore in line with, e.g., the study by Hollmann et al. (2012) [65], which observed significantly increased neural responses in dlPFC during the active regulation of the desire for unhealthy food. However, Kober et al. (2010) [70] also showed that a cognitive regulation of food and cigarette cravings increased neural activity in the dlPFC. These effects are explained by cognitive strategies that help diminish the craving by focusing on the long-term consequences of smoking. At the same time, it has been shown that a higher BMI is related to reduced neural activation in response to food images in the brain regions that are assigned to inhibitory control and self-control—i.e., the dlPFC, among others [18,67]. At this point we can only speculate, but one could think (and this should be investigated further) that the individuals with normal BMI, who showed increased neural activity, used the warning messages on the sweets to solve the conflict between external information (health warnings) and internal desire (tasty sweets) and regulated their desire better or more than the group with a high BMI.

Red nutrition labeling was thought to decrease the value of a product due to the negative information associated with the product, which was the case for the “Stop” condition [73]. To date, there has only been one other study that also used a stop sign (in black). The results did not show one of two different labels performing better than the other one in reducing the willingness to purchase for products that are high in sugar. The authors concluded that, in general, there is a high potential for some kind of front-of-package labels to reduce the sugar consumption, but the type of label might not be that important [74]; however, coloring may be a helpful guidance for consumers. This can be underlined by the reports of our participants, which showed that the majority of individuals actively perceived and remembered the health warning messages. Almost half of the statements we classified were about the perceived or associated general effect and effectiveness of the warnings and what the test persons felt when looking at them. This shows that the warning messages were able to provoke emotional reactions among the participants, which might be important to induce behavioral changes. However, whether these would be positive reactions (e.g., reduction of sugar content) or negative effects (e.g., reactance effects, habituation effects) cannot be conclusively determined at this time and should be investigated further.

We want to mention, further, that our study is missing additional behavioral measurements that would have allowed us to really validate the fNIRS findings with particular psychological processes, such as reward evaluation, social behavior consequences, or self-control. As such, we can only make first suggestions and state the mental processes that are related to these brain areas and that might be of interest for further research concerning the processing of health warning messages. Especially in areas such as neuroeconomic and consumer neuroscience, mental processes and underlying brain functions are much less understood. We aim to start to evolve into consumer behavior and consumers’ neural actions, which is why our results should be seen as first explorative insights on what might eventually be possible and what other studies can build upon. This might help to shed some light on the novel measurement technology fNIRS in the context of consumer behavior.

### Strengths and Limitations

This study has a number of strengths and thereby adds important value to the worldwide discussion about obesity prevention strategies. First of all, the measurement with fNIRS allowed us to examine a considerably larger number of persons, and thus considerably increased the generalizability of the neuroimaging results. In this case, we used photos of real products as stimuli and added graphical health warning messages that were adapted and placed in such a way that a realistic representation of the products remained. It should also be stressed that the recruited subjects were both normal-weight and overweight individuals, which increases the relevance of the results significantly, as potential preventive effects of warnings would probably have an effect on both groups—which is why it is crucial to investigate both groups at the same time.

This study also had limitations: Our trial number, with five runs and five pictures, was quite small. This might be one of the reasons why we did not detect strong effects in our data. To improve the reliability of the fNIRS signal, one could have increased the trial numbers, but we wanted to prevent the subjects’ attention from drifting away. Another shortcoming is that the headband we used cannot be fastened to the head as firmly and stably as, for example, a cap for the whole head, which can also be fastened under the subject’s chin. Therefore, for participants with thicker or darker hair, that absorbs more light, poor data quality can occur more quickly. This might explain the relatively high number of test persons that had to be excluded from our sample due to poor channel quality. Nevertheless, due to ethical and random sampling reasons, we did not screen people for their hair color. We also did not screen the participants for any eating disorders, which might have affected participants’ reactions to the food pictures. However, only two participants of our sample can be classified as underweight; only one reported a BMI lower than 16; so, if this affected the data, it did so only to very small parts.

Another limitation that needs to be considered is that we used a liberal significant threshold of 10% for the contrasts between the experimental and the control conditions. This was done because our conditions did not differ extremely, since we used the same product pictures for both experimental conditions and only the health warning messages differed, not leading to very strong reactions. However, in order to actually talk about, and give guidance for future studies in regard to, the differences between health warning label conditions, the interaction contrasts between the experimental condition and control condition are the really interesting ones, which is why we reported the results in this way.

All stickers were adjusted on “real-life” products to simulate real-life situations. Within, the sizes of the letters differed marginally but according to real market conditions. We used fixed margins for the text box, large enough for the text information to be easy to read on the product pictures. We did not control for this, so this could have affected the efficacy of the stickers.

## 5. Conclusions

This paper highlights the neural responses of participants during the perception of different health warning messages, as well as their differing effects on normal-weight and overweight people, measured with the innovative neuroimaging technology fNIRS. Our findings contribute to the growing body of literature about the potential of health warning messages as a possible intervention strategy to combat overweight. Stop signs and shocking images as additional graphical information for textual health warning messages were actively and emotionally remembered by the participants and seem to lead to increases in neural activation in the OFC, FPC, and dlPFC regions of the prefrontal cortex. Nevertheless, we cannot determine which effect they would have. Therefore, we see our results as a starting point for further research, especially concerning the effectiveness of a “Stop” symbol in reducing sugar consumption, because the symbol is widely known and thus already strongly anchored in people’s consciousness, but also concerning a further validation of fNIRS methodology against other elements of socio-empirical research in order to be able to connect the imaging findings to particular mental processes.
